# Editorial for the “Non-Coding RNAs in Human Health and Disease” Special Issue

**DOI:** 10.3390/genes16020211

**Published:** 2025-02-09

**Authors:** Sujay Paul

**Affiliations:** School of Engineering and Sciences, Tecnologico de Monterrey, Campus Queretaro, Av. Epigmenio González No. 500 Fracc. San Pablo, Querétaro 76130, Mexico; spaul@tec.mx

## 1. Introduction

Numerous non-coding RNA (ncRNA) species, including miRNAs, siRNAs, piRNAs, circRNAs, and lncRNAs, have displayed a substantial correlation with human diseases, and may serve as prospective targets for gene therapy and diagnostic biomarkers. In the present thematic issue, “Non-coding RNAs in Human Health and Disease”, significant articles detailing the discoveries about, and research on, ncRNAs that are involved in human disease development, and how they can be exploited for disease management, have been included. Numerous papers were submitted to this Special Issue; however, only eleven high-quality papers have ultimately been chosen for publication, after undergoing a rigorous review procedure in order to maintain the standards of the journal.

Coman et al. (2024) [1] examined the expression levels of miR-106a-5p and miR-148a-3p in blood plasma to distinguish between prostate cancer (PCa) and benign prostatic hyperplasia (BPH), emphasizing the downregulation of both miRNAs in PCa patients; the integrated analysis of the two miRNAs demonstrated enhanced diagnostic sensitivity relative to individual miRNA assessment, as validated by the ROC curve analysis. Likewise, Mazurek et al. (2024) [2] discussed the relationship between miR-5682 and the nutritional status of radiotherapy-treated male laryngeal cancer patients and, interestingly, the analysis of miR-5682 expression demonstrated a potential clinical utility in the selection of laryngeal cancer patients suffering from nutritional deficiencies developing as a consequence of radiotherapy-based treatment.

Lederer et al. (2024) [3] compared the performance of commonly used total RNA extraction methods for fecal microRNA isolation, and their study suggested that different isolation methods yield reliable and comparable miRNA expression results, hence endorsing the potential comparability and translational relevance of miRNA-based biomarker research in the future. Mensah-Bonsu et al. (2024) [4] identified several human microRNAs in Ebola virus infections, and demonstrated their potential roles in disease pathogeneses. Ishibashi et al. (2024) [5] demonstrated that myelin-specific microRNA-23a/b cluster deletion inhibits myelination in the central nervous system (CNS) during postnatal growth and aging. Their findings indicated that the deletion of myelin-specific miR-23a/b clusters in the CNS results in diminished myelin basic protein (MBP) and proteolipid protein (PLP) expressions, leading to myelin hypoplasia during postnatal development and aging.

The research findings of Nkechika et al. (2024) [6] indicated that certain miRNAs (miR-466c and miR-340) are involved in the palmitate-induced deregulation of gonadotropin-releasing hormone and its related transcription factors, which might facilitate the development of microRNA-based therapies for metabolism-related disorders, addressing the pathways that contribute to the diet-induced reproductive dysfunctions associated with obesity and infertility. MicroRNAs (miRNAs) are essential regulators during several phases of implantation, affecting the endometrial receptivity, embryonic development, and interaction between the embryo and endometrium. In this context, Cho et al. (2024) [7] predicted that two genes, namely AHCYL2 and BVES, coupled with their presumed regulator, miR-665, might function as biomarkers for diagnosing Recurrent Implantation Failure (RIF) in Korean women.

The clinical significance of the circular RNAs hsa_circ_0004018 and hsa_circ_0003570 in patients with hepatitis B virus-related hepatocellular carcinoma (HBV-HCC) is elusive. Kang et al. (2023) [8] sought to investigate the clinical relevance and prognostic value of these two circular RNAs in patients with HBV-related hepatocellular carcinomas (HBV-HCC). The relative expression profiles of hsa_circ_0004018 and hsa_circ_0003570 were assessed via quantitative real-time polymerase chain reactions on 86 paired tissue samples of hepatocellular carcinoma (HCC) and adjacent non-HCC tissues. They concluded that the combination of the high tissue expression of hsa_circ_0004018 and hsa_circ_0003570 is strongly associated with favorable clinical outcomes, and can be a novel prognostic marker in patients with HBV-HCC. In an interesting review article, Morando et al. (2024) [9] explored the role of microRNAs in HIV infection, while in another review article, Nakashima et al. (2024) [10] demonstrated the potential molecular mechanisms of alcohol use disorder with non-coding RNAs and gut microbiota for the development of superior therapeutic applications. In the final article (a brief report), Osorio-Pérez et al. (2023) [11] exhibited that the phytochemical Thymoquinone (TQ) potentially regulates the expression of crucial oncogenic and tumor suppressor (TS) miRNAs in the prostate (PC3) and colon cancer (HCT-15) cell lines. The findings of their study indicated that the phytochemical TQ possesses antiproliferative effects by reducing the proliferation of HCT-15 and PC3 cancer cells. Moreover, TQ modifies the expression of certain critical TS and oncogenic microRNAs, such as miR-200a-5p, miR-221-5p, miR-17-5p, miR-21-5p, and miR-34a-5p. The notable impact of miR-34a-5p, miR-221-5p, miR-17-5p, and miR-21-5p suggests their potential as therapeutic targets for the treatment of prostate and colon cancer, respectively.

In conclusion, this Special Issue on “Non-coding RNAs in Human Health and Disease” encompasses a variety of topics, and offers extensive insights to guide future research on the roles of different non-coding RNAs in human disorders and their therapeutic potential. We expect this Special Issue to inspire further research, resulting in enhanced comprehension and novel methodologies in the study of non-coding RNAs.
